# Editorial: Micro-and nanoplastics in agroecosystems: plant responses, soil interactions, and remediation strategies

**DOI:** 10.3389/fpls.2026.2017188

**Published:** 2026-10-21

**Authors:** Yuan Li, Yangzhou Xiang, Carmen Martín, Fulai Yan, You-Cai Xiong

**Affiliations:** 1 Natural Resources Institute Finland (Luke), Maaninka, Finland; 2 School of Geography and Resources, Guizhou Education University, Guiyang, China; 3 Departamento de Biotecnología-Biología Vegetal, Escuela Técnica Superior de Ingeniería Agronómica, Alimentaria y de Biosistemas, Universidad Politécnica de Madrid, Madrid, Spain; 4 College of Resources and Environment, Anhui Agricultural University, Hefei, Anhui, China; 5 State Key Laboratory of Herbage Improvement and Grassland Agro-ecosystems, College of Ecology, Lanzhou University, Lanzhou, China

**Keywords:** agricultural sustainability, agroecosystems, biochar, microplastics, nanoplastics, plant physiology, remediation strategies, soil health

While agricultural plastics can support crop production, their fragmentation poses a critical question: how do micro- and nanoplastics affect the functioning of the soil–plant system upon entering agricultural environments? Addressing this requires more than documenting particle presence or isolated plant responses. Plastic particles differ in size and composition, co-occur alongside other substances, and interact dynamically with soil conditions and management practices. This Research Topic brings together four experimental studies that examine plant responses, soil interactions, co-contamination and potential mitigation strategies. Together, they point toward an integrated research agenda and underscore why findings from controlled exposures should not be treated as field-scale risk estimates.

## Plant responses depend on both exposure level and environmental context

In a pot study of chicory, Song et al. tested polyethylene microplastics mixed into soil at mass ratios of 0.15–7.5%. Plant height and leaf width were not significantly affected at concentrations up to 1.5%, although fresh root weight declined in some lower-concentration treatments. At concentrations of ≥1.5%, leaf chlorophyll a and total chlorophyll declined significantly; higher concentrations also impaired aboveground growth. Thus, the absence of a significant response in some growth measures does not imply an absence of physiological effects. These findings highlight the value of assessing plant and soil responses together rather than relying on a single growth measure.

Particle characteristics matter as well. Using fluorescently labelled polylactic acid nanoplastics in a hydroponic pakchoi experiment, Zhao et al. observed their uptake into roots and movement to aboveground tissues, alongside biochemical responses. Their findings challenge the assumption that biodegradable polymers are inherently biologically innocuous once present as small particles. However, hydroponic exposure does not quantify how much of this movement occurs in agricultural soils or under field conditions. Soil-based studies are needed to link particle behaviour with realistic plant exposure scenarios.

## Plastics do not act in isolation

In a soybean study, Ren et al. examined polyethylene microplastics together with manganese, revealing scenario-dependent changes in soil nutrients, root traits, antioxidant activity and plant growth. The combined treatments did not support the simplistic assumption that two stressors always produce a more severe adverse response than either alone. This matters for agricultural research, as single-pollutant experiments may overlook interactions that modulate both exposure dynamics and biological outcomes. Future work should therefore specify the conditions under which co-contaminants amplify, mitigate or otherwise alter observed effects, rather than generalising from isolated combinations.

The question of mitigation management is equally important. In a pot experiment with *Amaranthus tricolor*, Khan et al. demonstrated that biochar partly alleviated adverse plant responses to combined polyethylene and oxytetracycline exposure while improving measured soil properties. However, biochar also modulated short-term soil CO_2_ emissions; it did not uniformly suppress them. An amendment should therefore be assessed across multiple dimensions, including plant performance, contaminant behaviour and soil carbon dynamics, rather than labelled successful based on a single favourable indicator. The study identifies a promising intervention to test further, not a field-validated remedy.

Across the four studies, a useful shift in perspective emerges: the central unit of analysis should be the managed soil–plant system, not the plastic particle alone. Three priorities follow. First, exposure studies need stronger links between the particles tested and those present in agricultural settings, with clear reporting of polymer, size, concentration and experimental conditions. Second, measurements should connect plant performance with soil properties and processes, particularly nutrient availability, microbial responses and carbon dynamics, without assuming that changes in a single measure establish a mechanism. Third, proposed interventions should be tested for trade-offs as well as benefits, and findings from pots or hydroponics should be evaluated under more realistic conditions before management recommendations are made. These are research priorities arising from the collection, not conclusions already demonstrated across agroecosystems.

For future studies, the strongest opportunity is to bring these strands together: trace agricultural plastic inputs, characterise exposure in soil, measure linked crop and soil outcomes, and evaluate whether management options improve the system. Such an approach can connect microplastic research with soil fertility, biogeochemistry and sustainable agricultural management while retaining the careful distinction between experimental evidence and practical application. The contributions to this Research Topic provide starting points for that work.

